# Elevation of circulating big endothelin-1: an independent prognostic factor for tumor recurrence and survival in patients with esophageal squamous cell carcinoma

**DOI:** 10.1186/1471-2407-8-334

**Published:** 2008-11-15

**Authors:** Wenjie Jiao, Jing Xu, Jinsheng Zheng, Yi Shen, Lesheng Lin, Jian Li

**Affiliations:** 1 Department of Thoracic Surgery, Peking University First Hospital, Beijing, PR China; 2 Department of Thoracic Surgery, Committee of Disciplinary Inspection, the Affiliated Hospital of Qingdao University Medical College, Qingdao, PR China; 3 Department of Thoracic Surgery, Wulanhot Hospital, Wulanhot, PR China

## Abstract

**Background:**

Endothelin(ET) axis plays a key role in many tumor progression and metastasis via various mechanisms such as angiogenesis, mediating extracellular matrix degradation and inhibition of apoptosis. However, there is limited information regarding the clinical significance of plasma big ET-1 levels in esophageal cancer patients. Circulating plasma big ET-1 levels were measured in patients with esophageal squamous cell carcinoma(ESCC) to evaluate the value of ET-1 as a biomarker for predicting tumor recurrence and patients survival.

**Methods:**

Preoperative plasma big ET-1 concentrations were measured by an enzyme linked immunosorbent assay(ELISA) in 108 ESCC patients before surgery, and then again at 1,2,3,10 and 30 days after curative radical resection for ESCC. The association between preoperative plasma big ET-1 levels and clinicopathological features, tumor recurrence and patient survival, and their changes following surgery were evaluated.

**Results:**

The preoperative plasma big ET-1 levels in ESCC patients were significantly higher than those in controls. And there was a significant association between plasma big ET-1 levels and disease stage, as well as invasion depth of the tumor and lymph node status. Furthermore, plasma big ET-1 levels decreased significantly after radical resection of the primary tumor and patients with postoperative recurrence had significantly higher plasma big ET-1 levels than that of patients without recurrence. Finally, the survival rate of patients with higher plasma big ET-1 concentrations (>4.3 pg/ml) was significantly lower than that of patients with lower level (≤ 4.3 pg/ml). Multivariate regression analysis showed that plasma big ET-1 level is an independent prognostic factor for survival in patients with ESCC.

**Conclusion:**

Plasma big ET-1 level in ESCC patients may reflect malignancy and predict tumor recurrence and patient survival. Therefore, the preoperative plasma big ET-1 levels may be a clinically useful biomarker for choice of multimodality therapy in ESCC patients.

## Background

The incidence of esophageal cancer shows a striking geographic variation in the world: a 20-fold variation is observed between high-risk China and low-risk western Africa[1]. Recently, advances in surgical techniques and peri-operative management significantly improved the outcome of patients with squamous cell carcinoma of the esophagus. However, the overall survival remains poor and the five year survival rate remains below 30 percent in patients with esophageal cancer after a curative esophagectomy[2-4]. Many results[5-8] demonstrated the prognosis in patients with esophageal cancer mainly depends on tumor stage, but other multiple factors, including age, gender, the size of tumor and some molecular markers, will influence tumor response to therapy. Accurate prognostic factor is essential for selecting patients who are suitable for combined-modality therapy. The use of circulating prognostic biomarkers is a convenient way to achieve the objective[9].

Endothelins(ETs), including ET-1, ET-2 and ET-3, are small 21-residue peptides[10]. There are at least two receptor subtypes, endothelin A receptors(ETAR) and endothelin B receptors(ETBR), belonging to the family of G-protein-linked receptors with seven transmembrane-spanning domains[11]. The ET-1 gene encodes a precursor peptide, preproendothelin-1, which is cleaved by a neutral endopeptidase to form proendothelin-1 or big ET-1. Due to a low circulating concentration and a short plasma half-life (about 1.5 min), measurement of plasma ET-1 concentrations has proven to be difficulty. Big ET-1 is a stable peptide with a plasma half-life of 30 minutes, making the measurement of plasma big ET-1 concentrations a sensitive indicator of endothelin system activation[12,13]. Recent studies [14-18] have suggested that ET-1 may play an important role in tumorigenesis, tumor progression and metastasis presumably by various mechanisms, including mitogenesis, inhibition of apoptosis, angiogenesis and mediating extracellular matrix degradation.

According to our previous study[19], ET-1 can increase the invasive ability of human esophageal cancer cells. However, it is unclear about prognostic significance of preoperative plasma big ET-1 in patients with ESCC. In this study, we evaluated: 1)plasma big ET-1 levels in ESCC patients and in healthy controls, 2) its correlation with clinicopathologic features, tumor recurrence and patient survival, and 3) the effect of surgery on plasma big ET-1 levels.

## Methods

### Patient Selection

The study population consists of 122 consecutive patients who underwent radical resection at our hospital between March of 2000 and August of 2002. All patients had been confirmed as esophageal squamous cell carcinoma by postoperative histopathologic assessment. Tumor stage was classified by the routine histopathologic assessment according to the UICC TNM staging system [20]. Patients who had received chemotherapy and/or radiotherapy before surgery were excluded from the study. Patients with co-morbid conditions that are associated with elevated ET-1, such as hypertension, cardiac failure, myocardial infarction and rheumatic diseases, were excluded. Moreover, 122 patients were asked about their habits of smoking and drinking. They were divided into three groups stratified by the number of cigarettes per day(cps) defined as non-smoker(have not smoked yet or very rarely tried to smoke), light-smoker(less than 20 cps) and heavy-smoker(more than 20 cps). And the patients were also divided into three groups stratified by ethanol intake levels defined as non-drinker (less than 1 g/day), light-drinker (1–50 g/day) and heavy-drinker (more than 50 g/day). Fourteen patients which were heavy smokers and/or drinkers were excluded in order to avoid potential interference. Out of a total of 122 such patients, 108 patients were the subjects of the present study. There were 78 men and 30 women with a median age of 64.5(range, 44–79) years.

The control group consisted of 82 age- and sex-matched healthy individuals(median age 65.3 [range, 40–72] years; 44 men and 38 women) without any evidence of disease. Moreover, we also collected data in 26 light-smokers, 35 heavy-smokers, 32 light-drinkers and 38 heavy-drinkers without ESCC and/or co-morbid conditions. The regional ethics committee approved the project. Patients were followed and the date and cause of death was recorded. The diagnosis of local/regional recurrence and distant metastases were confirmed by histology and/or imaging findings.

### Blood collection and assays

Peripheral venous blood samples were drawn into sterile glass tubes in the morning between 7 and 8 hours after an overnight fast. All blood samples was collected in EDTA specimen tubes, placed immediately into an ice bag and centrifuged at 2,000 rpm for 10 minutes at 4°C within 15 minutes of blood collection. Plasma was separated, aliquoted, and stored at -70°C until assay. Plasma samples were collected and stored on admission and 1,2,3,10 and 30 days following surgery. Plasma big ET-1 concentrations were measured using a one-step sandwich enzyme immunoassay kit (Biomedica, Vienna, Austria) in accordance with the manufacturer's protocol. The kit consisted of purified polyclonal antibody and monoclonal detection antibody highly specific for big ET-1. Big ET-1 binds to the pre-coated antibody and forms a sandwich with the detection antibody. Plasma big ET-1 concentrations were calculated by extrapolation from a standard curve. A separate standard curve was constructed for each ELISA batch. All standards and patient samples were analyzed in duplicate and the mean value was taken.

### Statistical analysis

The values of the plasma big ET-1 was written as mean ± standard deviation. T test and ANOVA were used to evaluate differences between multiple groups, unpaired and paired observations, respectively. Kaplan-Meier survival curves and the log rank test were used to analyses survival differences. Univariate and multivariate analyses (Cox's proportional hazard) of all clinicopathological variables were performed using SPSS version 10.1 (SPSS Inc, Chicago, IL, USA). A *p *value of less than 0.05 was considered significant.

## Results

### 1) Preoperative plasma big ET-1 levels

Preoperative plasma big ET-1 levels (4.70 ± 0.81 pg/mL) in patients with ESCC were significantly higher than those in controls (3.31 ± 0.78 pg/mL, *P *< 0.001), light-smokers(3.44 ± 0.71, P = 0.005) and light-drinker(3.36 ± 0.83, P = 0.003), heavy-smokers(4.12 ± 0.56, P = 0.020) and heavy-drinkers(3.86 ± 0.75, P = 0.011). Moreover, the levels in controls were significantly lower than those in heavy-smokers(P = 0.010) and heavy-drinkers(P = 0.038) without ESCC, and were no statistically different compared to the levels of light-smoker(P = 0.250) and light-drinker(P = 0.382).

The relationships between plasma big ET-1 levels and clinicopathologic variables are shown in Table 1. The correlation between plasma big ET-1 levels and the invasion depth of the tumor was statistically significant by ANOVA. Patients with tumors penetrating the muscle (T3) had significantly higher plasma big ET-1 levels when compared to those with tumors limited to the mucosa, submucosa or muscle(T1/2). And patients with lymph node metastasis had significantly higher big ET-1 levels when compared with those without metastatic disease. Moreover, there was a significant correlation between plasma big ET-1 levels and disease stage, with higher big ET-1 levels detected as the disease stage increased.

**Table 1:** Correlation between preoperative plasma big ET-1 levels and clinicopathologic variables in ESCC patients

Characteristic	Number of patients (n)	Preoperative plasma big ET-1 levels (pg/ml)	P value
Gender			
Male	78	4.62 ± 0.72	0.285
Female	30	4.85 ± 0.63	
Age, y			
≤ 60 yrs	41	4.67 ± 0.55	0.876
>60 yrs	67	4.72 ± 0.86	
Smoking			
Light-smoker	42	4.78 ± 0.39	0.386
Non-smoker	66	4.65 ± 0.64	
Drinking			
Light-drinker	35	4.73 ± 0.27	0.674
Non-drinker	73	4.68 ± 0.50	
Site of tumor			
upper	12	4.75 ± 0.91	0.763
Middle	57	4.70 ± 0.57	
Lower	39	4.69 ± 0.83	
Tumor differenciation			
Well	30	4.61 ± 0.54	0.354
Moderate	48	4.64 ± 0.68	
Poor	30	4.88 ± 0.47	
Tumor class			
T1	33	4.42 ± 0.61	0.001
T2	38	4.60 ± 0.34	
T3	37	5.01 ± 0.84	
T4	0	0	
Lymph node metastasis			
N0	63	4.45 ± 0.59	<0.001
N1	45	5.06 ± 0.63	
Stage			
I	28	4.41 ± 0.52	0.003
II	48	4.68 ± 0.76	
III	32	5.25 ± 0.82	
IV	0	0	

There were no significant associations between plasma big ET-1 levels and gender, age, tumor location and degree of differentiation.

### 2) The effect of surgery on plasma big ET-1 Levels

**Figure 1:**
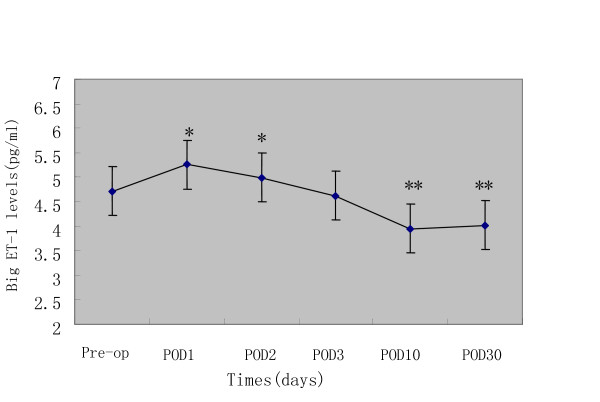
**Changes in plasma big ET-1 levels following esophagectomy.** *elevated on POD1 and POD2(P < 0.001) and **reduced on POD10 and POD30(P < 0.001) compared with preoperative levels.

The effect of surgical resection of the tumor was evaluated by sequential measurement of plasma big ET-1 levels before surgery and at 1,2,3,10 and 30 postoperative days(POD) following surgery. Radical resection including subtotal esophagectomy and removal of the regional lymph nodes (two-field lymph node dissection) was performed in all 108 patients. The plasma big ET-1 levels on the first and second postoperative day increased significantly compared with preoperative levels in 108 patients, but decreased on the 3rd POD with no significant difference compared to the pre-operative levels. And then, there was a subsequent decrease with the 10 day postoperative levels being significantly lower than preoperative levels. The plasma big ET-1 levels at 30 days remain stable when compared with 10 days postoperative values(P = 0.273).(Figure 1)

### 3) The overview of follow-up

All patients lived at least 30 days after surgery, and were followed-up using a standard protocol after discharge from the hospital. The mean follow-up period was 34.2 months (7~60 months). Cancer-specific survival was calculated from the date of operation until date of death or last follow-up. Survival was censored for patients who died without disease progression. During the study, 63 patients died from ESCC progression (including 19 patients who developed local/regional recurrences and 44 patients who developed distant recurrences) and 45 patients remained alive, 7 of them with documented tumor recurrence.

### 4) Correlations between preoperative plasma big ET-1 levels and patients survival

**Figure 2:**
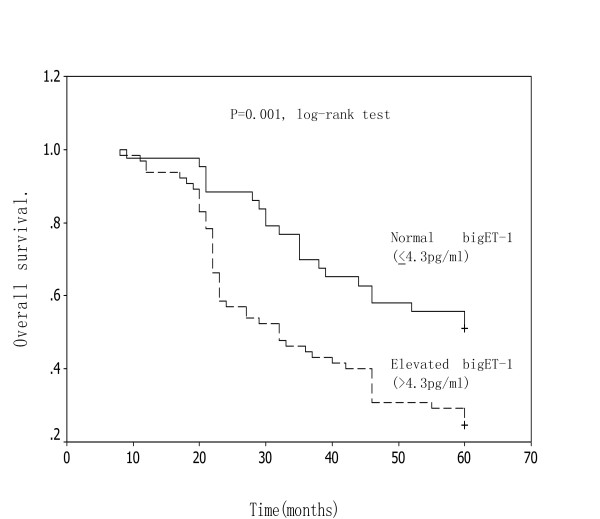
**Kaplan-Meier survival curve in relation to preoperative plasma big ET-1 levels in patients with ESCC.** The overall survival of patients with elevated big ET-1 levels was significantly lower than that of patients with normal levels(log-rank test P = 0.001).

Because 4.3 pg/mL was the upper limit of plasma big ET-1 concentrations in healthy controls(2.9~4.3 pg/mL), the value was regarded as a threshold according to previous report[9,12]. Elevated plasma big ET-1 levels were found in 71(65.7%) patients. The overall survival rate of patients with higher plasma big ET-1 concentrations (>4.3 pg/ml) was significantly lower than that of patients with lower level(≤ 4.3 pg/ml).(Figure 2)

Prognostic variables evaluated in a univariate analysis included age, gender, tumor location, differentiation, T class, lymph node status, TNM stage and preoperative plasma big ET-1 level. (Table 2) Multivariate regression analysis identified four variables, T class, lymph node status, TNM stage and preoperative plasma big ET-1 concentration as having independent prognostic value for overall survival. (Table 3)

**Table 2:** Univariate analysis for predictors of overall survival in patients with ESCC

Variable	Hazard Ratio	95% Confidence Interval	P value
Age (>60 vs. ≤ 60)	0.834	0.512–1.357	0.834
Gender (male vs. female)	1.183	0.727–1.924	0.499
Site of tumor (upper vs. middle vs. lower)	1.310	0.916–1.872	0.139
Differenciation (well vs. moderate vs. poor)	1.084	0.799–1.470	0.604
Tumor class (T1 vs. T2 vs. T3 vs. T4)	2.763	1.632–4.854	0.008
Lymph node status (No vs. N1)	2.978	1.541–3.647	0.010
Stage (I vs. II vs. III vs. IV)	3.037	1.451–4.860	<0.001
Plasma big ET-1 levels (normal vs. elevated)	2.494	1.302–3.806	0.002

**Table 3:** Multivariate analysis for predictors of overall survival in patients with ESCC

Variable	Hazard Ratio	95% Confidence Interval	P value
Tumor class	2.878	1.805–4.576	0.010
Lymph node status	2.921	1.951–3.988	0.015
Stage	3.083	1.259–5.731	0.002
Plasma big ET-1 levels	2.629	1.375–4.054	0.003

### 5) Correlations among preoperative plasma big ET-1 levels, tumor recurrence and survival

**Figure 3:**
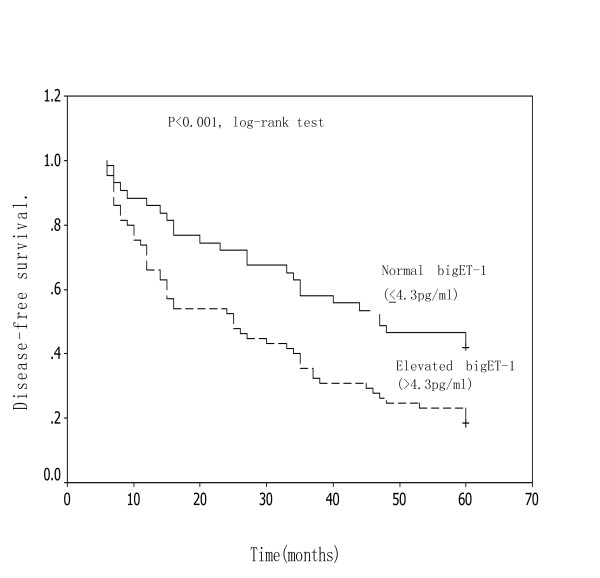
**Kaplan-Meier survival curve in relation to preoperative plasma big ET-1 levels in patients with ESCC.** The disease-free survival of patients with elevated big ET-1 levels was significantly lower than that of patients with normal levels(log-rank test P < 0.001).

Patients with recurrence had significantly higher preoperative plasma big ET-1 levels than patients without recurrence(5.42 ± 0.66 pg/ml versus 4.46 ± 0.48 pg/ml, P = 0.002). About 78.6%(55/70) patients with tumor recurrence remained high plasma big ET-1 level (>4.3 pg/ml) when tumor recurrence were found. Moreover, in the patients with tumor local-regional and/or distant recurrence, the disease-free survival rate of patients with elevated plasma big ET-1 concentrations (>4.3 pg/ml) was significantly lower than that of patients with lower level(≤ 4.3 pg/ml).(Figure 3)

## Discussion

To date, pathologic stage is the most valuable single prognostic attribute in esophageal cancer patients. In the present study, we evaluated preoperative plasma big ET-1 levels in healthy controls and in ESCC patients using a validated ELISA. Our study shows a marked difference between preoperative plasma big ET-1 concentrations in ESCC patients when compared with age and sex-matched healthy controls, with a significant association between these levels and tumor invasion depth, lymph node status and TNM stage. Univariate and multivariate survival analysis confirmed that pre-operative plasma big ET-1 level were an independent predictor of survival in patients with ESCC.

Some findings[21-24] suggest that ET-1 may act as an autocrine or paracrine growth factor mediating through its receptor, ETAR or ETBR. It is well established that endothelin axis plays an important role in a series of events relating with tumor development, including mitogenesis[25,26] and escape from apoptosis[27,28]. Moreover, ET-1 expression has been linked to induction of endothelial cell growth, angiogenesis[29-31] and epithelial-to-mesenchymal transition[32-34] resulting in an increased invasiveness and metastases of some tumors. ET-1 expression in ESCC has been associated with its prognostic significance[35]. Our findings are in agreement with the observations and correlate preoperative plasma big ET-1 levels with cancer-specific survival. Based on our results, we identified elevated level of preoperative plasma big ET-1 could act as a marker of aggressive disease and poor survival in ESCC. We speculate that increased plasma big ET-1 level may indicate more tumor load and/or unidentified micrometastatic disease, predicting a poor survival in patients.

In our study, the mean plasma big ET-1 levels were markedly increased on the 1st and 2nd POD in ESCC patients. The phenomenon may partly contribute to a compensatory response to surgical stress, for example, the reduction of local blood flow in nonvital organs so as to increase the blood flow in vital organs[36]. Several prior reports[37-39] suggest that tissue damage, ischemia and surgical stress during operation could raise circulating ET-1 levels, and the elevated ET-1 levels related to surgery frequently peak between 1 and 12 hours in the immediate post-operative period and declined gradually thereafter. On the 3rd POD, the plasma big ET-1 levels declined, but were no statistically different compared to the pre-operative levels. It implies that the increasing levels of big ET-1 as response to surgical stress were lessen. On the 10th and 30th POD, these levels declined significantly compared to pre-operative levels. Ferrari-Bravo[40] and Teng[41] also reported that postoperative plasma big ET-1 level markedly decreased compared with preoperative concentrations in patients with gastric carcinoma, and concluded that ET-1 may be secreted by the cancer cell and the ET-1 concentration will fall in when the tumor is removed. These findings[35,40,41], along with our results, suggest that plasma big ET-1 might be able to be used as a measure of surgical completeness.

During the study, preoperative plasma big ET-1 levels were an important independent prognostic factor for postoperative tumor recurrence in patients with ESCC. Those patients with recurrence had significantly higher preoperative plasma big ET-1 levels than patients without recurrence and the overall and disease-free survival rate of patients with elevated plasma big ET-1 concentrations was significantly lower than that of patients with normal level. Our findings suggest that increased plasma big ET-1 level may indicate unidentified micrometastatic disease, and may be used for predicting tumor recurrence in a proportion of patients with ESCC. Postoperative tumor recurrence is not uncommon even in patients undergoing a curative resection for localized resectable esophageal cancer. Micrometastatic tumor cells to either lymph nodes or distant organs which cannot be detected by preoperative imaging techniques may be attributed to such tumor distant failure. It is logical to expect a decline in tumor recurrence with the use of multiple therapy in part of high-risk patients with ESCC. To sterilize occult micrometastases in the distant organs, adjuvant chemotherapy either alone or in combination with radiotherapy is now commonly performed in patients with esophageal cancer either before or after an esophagectomy in an attempt to improve both disease-relapse control and long-term survival[42].

## Conclusion

In conclusion, this study has demonstrated elevated plasma big ET-1 levels in ESCC patients when compared with normal controls. Preoperative plasma big ET-1 concentration decreases significantly following radical resection of the primary tumor and is an independent prognostic factor for patient survival. Preoperative plasma big ET-1 concentration can be used for predicting tumor recurrence and may be a clinically useful biomarker for choice of multimodality therapy in ESCC patients.

## Competing interests

The authors declare that they have no competing interests.

## Authors' contributions

JW carried out the design of the study, the ELISA studies, follow-up and drafted the manuscript. XJ and ZJ participated in the design of the study and performed the statistical analysis. SY, LL and LJ participated in its design and coordination. All authors read and approved the final manuscript.

## Pre-publication history

The pre-publication history for this paper can be accessed here:


